# A realist review of mobile phone-based health interventions for non-communicable disease management in sub-Saharan Africa

**DOI:** 10.1186/s12916-017-0782-z

**Published:** 2017-02-06

**Authors:** Daniel Opoku, Victor Stephani, Wilm Quentin

**Affiliations:** Department of Healthcare Management, Technische Universität Berlin, Straße des 17. Juni 135, Sekretariat H80, 10623 Berlin, Germany

**Keywords:** mHealth, Mobile phone, Non-communicable diseases, Chronic diseases, Sub-Saharan Africa, Realist review, Health policy

## Abstract

**Background:**

The prevalence of non-communicable diseases (NCDs) is increasing in sub-Saharan Africa. At the same time, the use of mobile phones is rising, expanding the opportunities for the implementation of mobile phone-based health (mHealth) interventions. This review aims to understand how, why, for whom, and in what circumstances mHealth interventions against NCDs improve treatment and care in sub-Saharan Africa.

**Methods:**

Four main databases (PubMed, Cochrane Library, Web of Science, and Google Scholar) and references of included articles were searched for studies reporting effects of mHealth interventions on patients with NCDs in sub-Saharan Africa. All studies published up until May 2015 were included in the review. Following a realist review approach, middle-range theories were identified and integrated into a Framework for Understanding the Contribution of mHealth Interventions to Improved Access to Care for patients with NCDs in sub-Saharan Africa. The main indicators of the framework consist of predisposing characteristics, needs, enabling resources, perceived usefulness, and perceived ease of use. Studies were analyzed in depth to populate the framework.

**Results:**

The search identified 6137 titles for screening, of which 20 were retained for the realist synthesis. The contribution of mHealth interventions to improved treatment and care is that they facilitate (remote) access to previously unavailable (specialized) services. Three contextual factors (predisposing characteristics, needs, and enabling resources) influence if patients and providers believe that mHealth interventions are useful and easy to use. Only if they believe mHealth to be useful and easy to use, will mHealth ultimately contribute to improved access to care. The analysis of included studies showed that the most important predisposing characteristics are a positive attitude and a common language of communication. The most relevant needs are a high burden of disease and a lack of capacity of first-contact providers. Essential enabling resources are the availability of a stable communications network, accessible maintenance services, and regulatory policies.

**Conclusions:**

Policy makers and program managers should consider predisposing characteristics and needs of patients and providers as well as the necessary enabling resources prior to the introduction of an mHealth intervention. Researchers would benefit from placing greater attention on the context in which mHealth interventions are being implemented instead of focusing (too strongly) on the technical aspects of these interventions.

**Electronic supplementary material:**

The online version of this article (doi:10.1186/s12916-017-0782-z) contains supplementary material, which is available to authorized users.

## Background

In sub-Saharan Africa (SSA), the prevalence of non-communicable diseases (NCDs) is increasing rapidly [1], placing a growing burden on already weak health systems in the region [2, 3]. At the same time, the use of mobile phones is continuously rising, expanding the opportunities for the implementation of mobile phone-based health interventions (mHealth interventions) [4–6]. The World Health Organization (WHO) has proposed the further development and more widespread use of mHealth interventions for the prevention, management, and treatment of NCDs and their risk factors as part of its Global Action Plan for the prevention and control of NCDs [7].

In fact, mHealth interventions are increasingly used in low- and middle-income countries, including those in SSA [8]. Three recent systematic reviews, two specifically focusing on the use of mHealth interventions for the care and management of NCDs in SSA [6] and in developing countries [9], and the other looking more broadly at the use of mHealth interventions against chronic diseases in developing countries [10], found that the included studies generally reported positive outcomes. However, the authors also noted that there was insufficient evidence to support the scale-up of mHealth interventions because there were only five studies from SSA countries [6] and only nine studies from developing countries [9, 10]. In addition, the authors highlighted that further research was needed to better understand the causal pathways linking mHealth to improved care for patients with NCDs [6].

Traditional systematic reviews, which are often focused on randomized controlled trials, usually do not allow one to uncover causal pathways or to identify contextual mechanisms that may explain whether, why, and how interventions might work [11]. Realist reviews have emerged as an alternative method for systematic reviews, aiming to provide answers for policy makers about the causal mechanisms that link context, intervention, and outcomes [12].

Understanding these mechanisms is particularly relevant for complex interventions, such as mHealth interventions, which are implemented in vastly different healthcare settings (varying from rural communities [13, 14] to major university hospitals [15]), use various functions of mobile phones (from text messaging [16–18] to picture transmission [19]), target widely different health conditions (from skin lesions [20] to maternal health [21, 22]), and are put to use by persons with very different backgrounds, behaviors, skills, and beliefs [23–25].

This review aimed to understand how, why, for whom, and in what circumstances mHealth interventions contribute to improved treatment and care for patients with NCDs. More precisely, the first question (“how?”) that the review aimed to answer was: What is the specific contribution that mHealth makes to patient treatment and care? As the review proceeded, it became clear that the main contribution of mHealth interventions is that they facilitate (remote) access to previously unavailable — and often specialized — services. Therefore, the objective of this review was to answer the following specific questions: (1) What are the causal mechanisms (”why?”) that explain if an mHealth intervention facilitates access to care? (2) How do patient and provider characteristics (”for whom?”) influence these mechanisms? (3) What is the influence of contextual factors (”what circumstances?”) on these mechanisms?

## Methods

This review followed guidelines for realist reviews [11, 12, 26, 27] because the research questions could not be answered using more traditional forms of systematic reviews. Realist reviews focus on identifying (middle-range) theories, which can provide guidance to the available literature. These theories then help us to understand the mechanisms that explain why an intervention has worked in one context but not in another. However, such Context-Mechanism-Outcome (C-M-O) relationships identified in realist reviews do not imply that a specific context will *always* lead to a specified outcome. Instead, realist reviews assume that outcomes are the result of choices made by individuals whose interactions are influenced by the intervention and by the context of implementation [12, 26, 27]. (See Table 1 for the operational definition of the C-M-O model of hypotheses adapted in this review.)

**Table 1:** Operational definition of the C-M-O model of hypotheses adapted in this review

C-M-O	Operational definition
Context	This is defined as the prevailing conditions and circumstances within which patients and/or healthcare providers behave or decide to use mobile phone-based health interventions for the treatment and care of non-communicable diseases in sub-Saharan Africa. For example: *-* Patient/provider predisposing characteristics (age, gender, etc.) *-* Patient/provider needs *-* Patient/provider enabling resources
Mechanism	The factors or active ”ingredients” of a mobile phone-based health intervention which directly/indirectly influence both intended and unintended health outcomes and/or outputs of the treatment and care of non-communicable diseases in a well-defined context in sub-Saharan Africa. For example: - How easy to use the patients and healthcare providers find the mobile technology involved in the intervention - How useful patients and healthcare providers perceive the mHealth intervention to be over alternative programs and forms of accessing healthcare
Outcome	This constitutes the sustained use of mHealth interventions and — in turn — better patient access to care

### Scoping the literature and searching for relevant studies

An initial scoping review was conducted to identify candidate theories (see below) and to obtain a broad overview of the available literature on mHealth interventions aiming to improve treatment and care for patients with NCDs in SSA. Following this initial search, the review question was progressively refined to focus more specifically on the contribution of mHealth to facilitating access to previously unavailable care.

A search strategy was developed, using various combinations of the following search terms: “mHealth”, “non-communicable diseases”, and “sub-Saharan Africa”. PubMed, Cochrane Library, Web of Science, and Google Scholars, were searched and re-searched from March to May 2015. (Additional file 1 provides details of the search strategies developed for the four databases.) In addition, a hand search was performed of the *Journal of Telemedicine and Telecare*, the *Journal of Telemedicine and e-Health*, and of reference lists of screened studies and existing reviews.

### Inclusion and exclusion criteria

The review included various study designs (randomized controlled trials, mixed methods, and qualitative interview studies) and publication types (peer-reviewed articles, gray literature, and other forms of research reports). Titles, keywords, and abstracts were screened by the corresponding author (DO) to identify relevant studies based on a set of inclusion criteria developed during the initial scoping review. A second reviewer (VS) also independently screened retrieved studies. If there was disagreement between reviewers, studies were retained for full-text screening. The following inclusion criteria were applied: (1) studies took place in sub-Saharan Africa (i.e., in at least one of the 47 countries in the WHO African region), (2) interventions relied on the use of (mobile) phones, (3) studies focused on NCD-related treatment and care, and (4) studies provided an evaluation of the relationship between the intervention and NCD care. No language restrictions or time limits were applied.

Full-texts of 126 studies were retrieved and independently screened by DO and VS. At this stage, studies were excluded if interventions were based on phones and not primarily on mobile phones. In case of doubts, corresponding authors of studies were consulted for clarification. Studies were also excluded if they did not report results of (clinical) outcomes and/or an assessment of the intervention by patients, professionals, or proxies (e.g., relatives or guardians). In case of disagreements between DO and VS on the eligibility of studies, these were resolved by WQ.

### Identifying candidate theories

During the initial scoping review, a number of candidate theories with potential explanatory value for mHealth interventions were explored. The identified theories and models included the Middle-Range Theory of Self-Care of Chronic Illness [28], the Theory of Reasoned Action/Theory of Planned Behavior [29], Rosenstock’s Health Belief Model [30], Andersen’s Behavioral Model of Health Services Utilization [31, 32], Young’s Choice-Making Model [33], and Davis’s Technology Acceptance Model [34, 35]. (See Additional file 2 for the reasons of inclusion/exclusion.)

Following discussions within the review team, Andersen’s Behavioral Model of Health Services Utilization was retained because it could potentially provide insights into the mechanisms linking contextual and individual level factors with improved access to care. According to Andersen’s model, peoples’ decisions to use (or access) healthcare services are determined by three main factors: (1) predisposing characteristics (e.g., age, health beliefs), (2) enabling resources (e.g., availability of providers), and (3) need (e.g., burden of disease) [32].

As the review proceeded, Davis’s Technology Acceptance Model was found to provide additional insights into mechanisms that are important for explaining improved access to care through mHealth interventions. Davis’s Technology Acceptance Model posits that the use and acceptance of technology is determined by two factors: *perceived usefulness* and *perceived ease of use*. According to Davis’s theory, health professionals will perceive a technology to be useful if they believe that it will help them to do a better job, and they will perceive a technology to be easy to use if they believe that it can be used without effort [35].

### Data extraction, analysis, and synthesis

Two data extraction templates were developed using Excel to collate information on the included studies for analysis and synthesis. One template was used to summarize the characteristics of included studies (author(s), year of publication, title, study design, and country where the study took place). The other template for results and synthesis mainly contained information on the (type of) intervention, modality of interaction, outcome/outputs, and the five categories of the theoretical model: *predisposing characteristics, enabling resources, need, perceived usefulness, and perceived ease of use*.

The data synthesis involved team discussions in relation to whether the information extracted was rightly placed in the various domains and adjusted accordingly. Common themes were highlighted, examined, and refined in the light of their theoretical contributions. This involved classifying findings from different studies into the categories of the theoretical model in order to understand the Context-Mechanism-Outcome (C-M-O) relationship. For example, if a study reported that older age groups were more likely to make use of an intervention because they found it more useful than younger age groups, this finding was classified into the category of a *predisposing characteristic* that leads to *perceived usefulness*.

## Results

### Search results and study characteristics

**Fig. 1:**
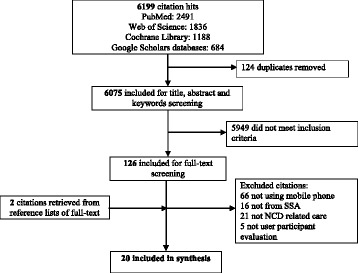
Flowchart displaying the search results and the screening process for the primary studies

A total number of 6201 citations were retrieved, out of which 6181 were excluded after the appraisal process displayed in Fig. 1. The raw inter-rater agreement between DO and VS was 97% (123/126) after full-text screening. Additional file 3 provides information on key characteristics of the 20 included studies. The studies were published between 2005 and 2015, and presented information on 18 interventions in various areas of care (dermatology, mental healthcare, cancer, diabetes, and hypertension).

### The contribution of mHealth to improved treatment and care for patients with NCDs

The main contribution of mHealth interventions to improved treatment and care for patients with NCDs in SSA countries is that they facilitate (remote) access to previously unavailable — and often specialized — services. In fact, almost all included studies highlighted this characteristic feature of mHealth interventions [20, 36–51].

However, the configuration of mHealth interventions differed considerably across settings, concerning involved actors and the mechanisms through which they facilitated access to care. In 12 studies, mHealth interventions essentially consisted of mobile phone-based consultations between two healthcare providers, where a specialized provider could be reached by another provider, thus indirectly improving patient access to specialized care [36, 38–43, 45, 47–49, 52]. In 8 studies, mHealth interventions connected a patient to a provider, thus directly facilitating patient access to (professional) care [20, 37, 46, 50, 51, 53–55].

Participating patients or providers usually required only a few days of training on how to use the mobile technology (such as the mobile phone and its application software) and the consultation procedures [41, 43, 46–48, 50, 55]. An important feature of most mHealth interventions was that interactions between participants usually took place on the basis of standardized information exchange protocols [36, 39, 40, 42, 43, 45–47, 49, 52, 54]. These protocols helped to establish the purpose of the consultations and contributed to systematically ascertaining symptoms, diagnoses, and treatment. (See Additional file 3 for further details.)

### From candidate theories toward a framework for understanding mHealth interventions

During the early stages of the review, Andersen’s model and his conceptualization of predisposing characteristics, enabling resources, and need helped to focus the analysis on the role of the context in explaining why mHealth interventions contribute to improved access for some patients and in some areas but not in others. However, as the review proceeded, it became increasingly clear that the context has only an indirect influence on access to health services facilitated by mHealth interventions. At this stage, Davis’s Technology Acceptance Model and his conceptualization of perceived usefulness and perceived ease of use contributed to understanding the mechanisms that link the context to improved mHealth based access to healthcare.

**Fig. 2:**
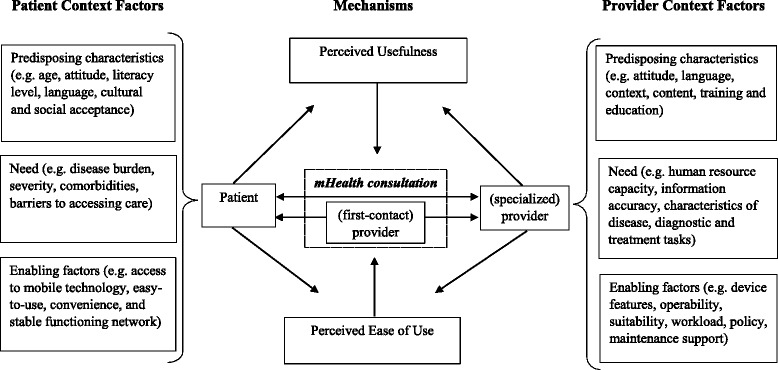
Framework for understanding the contribution of mHealth interventions to improved access to care for patients with NCDs in sub-Saharan Africa

The two models of Andersen and Davis were then integrated into a framework for understanding the contribution of mHealth interventions to improved access to care for patients with NCDs in SSA. The framework is illustrated in Fig. 2 and shows that mHealth consultations take place either between a patient and a provider or between two providers with one provider facilitating patient access to another provider with certain specialized skills. The most important patient context factors (predisposing characteristics, enabling factors, and need) are shown on the left-hand side of the figure, while the most important context factors for (specialized) providers are shown on the right-hand side. For providers facilitating access between patients and (specialized) providers, context factors are sometimes more similar to those of patients and sometimes more similar to those of (specialized) providers.

In the center of the figure, arrows indicate the C-M-O relationship: The context factors influence the perceptions of patients and providers concerning how useful they find the mHealth intervention in comparison with other forms of service delivery, such as traditional face-to-face contacts or alternative computer-based telemedicine. Similarly, these factors also influence the perceived ease of use of mHealth in comparison with other options for service delivery. If interventions are perceived to be useful and easy to use, this will lead to the sustained use of mHealth interventions and — in turn — to better patient access to care (see Table 1).

### Main findings from the literature

Table 2 summarizes the main findings from the literature, using the framework described above. It specifies separately for patients, (first-contact) providers, and (specialized) providers, what predisposing characteristics, enabling resources, and needs influence the perceived usefulness and the perceived ease of use.

**Table 2:** Detailed classification of evidence supporting the framework for understanding why, for whom, and in what circumstances mHealth interventions work in sub-Saharan Africa

	Patient		(First-contact) provider^a^		Specialized provider^b^	
Mechanism	Perceived usefulness	Perceived ease of use	Perceived usefulness	Perceived ease of use	Perceived usefulness	Perceived ease of use
Context						
Predisposing characteristics	• Cultural and social acceptance (familiarity/usage of mobile technologies) [38, 40, 41, 46, 53, 55] • Positive attitude (motivated, self-empowered, activeness) [38, 55] • Age group (middle/older) [51] • Language of communication (language of locality) [46, 54, 55]	Suitability and simplicity for: • (Older) age group [55] • (Low) literacy, educational levels [50, 55] • (Poor) socio-economic backgrounds [50] • Not physically active [50]	• Positive attitude (enthusiastic, motivated, empathetic, interest, dedication, volunteer) [38, 40, 52] • Prerequisite knowledge (to provide adequate information) [47]	• Simple, relevant, combination of local content and language (interface) [42]	• Positive attitude (positive perception and trust of new technology) [52] • Basic knowledge (about the technology) [52] • Fluency in language of locality [46, 54]	• Accessible location of technical support (in-country or local software developers) [52] • Understandable language of communication (among users and software developers) [52]
Need	• Disease severity and comorbidities [20, 51, 55] • Barriers to accessing care or information (not affordable, easily, promptly, quality and/or appropriate, limited, long-distance travel, travel cost, waiting time, delaying, presenting late) [20, 42, 45, 46, 48–50, 52, 53, 55]	-	• Lack of capacity to provide needed care (limited training/education, decision-making power/support, point-of-care clinical information, specialized care, specialty referral systems) [36, 38–40, 47, 52] • Barriers to reporting and accessing supervision [37, 40–42, 47, 51] • Need to follow guidelines [50, 54, 55]	-	• Lack of human resources (limited specialists, trained or skilled personnel, unequal distributions of professionals, over-burdened workload) [20, 36, 38–41, 43, 45, 47] • Lack of necessary systems and infrastructure (health facility, referral system, transport) [38] • Lack of accurate information [46, 47] • Task shifting to achieve early intervention and low costs of care [42, 43, 49, 54]	• Characteristics of disease conditions (extent, severity) [36, 43] • Characteristics of diagnostic and treatment tasks (feasibly assess/examine, freely question patient, probe for additional information, conduct special tests) [43, 47, 49]
Enabling resources	• Access to mobile phone [37, 45, 46, 50, 53–55] • Access to mobile technology infrastructure [45, 48, 52, 55] • Affordability of services [50, 54, 55] • Convenience, privacy, autonomy, reduced time and travel cost [20, 43] • Service/program awareness [38, 40] • Assistance/support (spouse, partner, friend, family member) [51]	• Familiar and easy-to-use mobile technology (SMS, icons) [53, 55] • Maintenance (phone recharge, repair, durability, portability) [37, 55]	• Access to phone [41, 45] • Telecommunication networks (functioning, stable, accessible, available, low-cost) [36, 39, 42, 47] • Basic infrastructural resources (information, good roads, ambulance services) [41, 47, 52] • Operating funds and logistics (availability) [38, 40, 52] • Policy and sustainability (to avoid strike actions, staff turnover rate) [40, 52] • Continuous training (workshops) and sensitization [47, 52] • Tolerable burden of workload [40]	• Easy portability and operability (direct, instant, immediate) [36, 39] • Phone features (quality camera, smartphones) [36, 41] • Maintenance support (equipment/SIM card/mobile device failure, sporadic power outages, battery power problem, software bugs, theft, medical technology) [45, 52]	• Access to phone networks (in underserved communities) [20] • Tolerable burden of workload [46, 47] • Incentives (payment) [47, 55] • Policy (network or data protection, liability, consent, confidentiality, phone usage, staff job descriptions) [43, 45, 51, 52]	• Phone features (photograph, picture quality, video functionality, interface, text messaging, appropriate screen, zoom, long-lasting battery) [36, 41, 43, 45, 47, 51] • Suitability and equivalence to existing care processes (face-to-face care, assess nonverbal behaviors) [41, 49, 51, 54]

^a^(First-contact) provider = the referring/consulting healthcare provider, usually in a provider-to-provider mHealth consultation

^b^Specialized provider = the consultant specialist or experienced healthcare provider whose expertise is being sought in mHealth consultations

Source: authors’ own compilation based on the findings of the included studies in this review

#### Predisposing characteristics

For patients, the most important predisposing characteristic associated with the perception that a mHealth intervention was more useful than an alternative was the patients’ cultural and social acceptance of the mobile technology, which involved familiarity with the technology in the community and absence of negative myths [38, 40, 41, 46, 53, 55]. Other important predisposing characteristics of patients included positive attitudes toward the intervention and the ability to communicate in a comfortable language (see Table 2). Similar predisposing characteristics were also reported for providers, i.e., positive attitudes [38, 40, 52], fluency in the language of the locality [46, 54], and sufficient training to use the technology [47, 52].

For both patients and (specialized) healthcare providers another important predisposing characteristic associated with the perception that mHealth was useful was source confidentiality [20, 39, 40, 49, 51]: Healthcare providers have to be confident that the information received via the mobile phone is accurate, and patients have to trust the (specialized) provider on the line in order to perceive the intervention as useful.

The perceived ease of use of an mHealth intervention depended most importantly on the predisposing characteristic that patients and providers were able to understand the language (see Table 2). In addition, studies reported that mHealth interventions have to be specifically designed to be easy to use for particular groups of patients, such as older age groups [55], or people with low educational levels [50, 55] or poor socio-economic backgrounds [50]. First-contact providers found mobile phone technologies easy to use if they were simple, relevant, and essentially combined local content and language [42]. Specialized providers’ perception of ease of use was influenced by the accessibility of technical support, especially when there was the need to identify and solve technical problems such as software bugs [52].

#### Need

Patient needs were found to be particularly important factors influencing the perceived usefulness of mHealth interventions. If patients faced access barriers such as long travel times, waiting times, and high travel costs, mHealth interventions were perceived to be useful [20, 42, 45, 46, 48–50, 52, 53, 55]. Furthermore, three studies found that sicker patients were more likely to use the interventions, possibly because they found it easier to use the mHealth interventions rather than, for instance, walk to a provider [20, 51, 55].

The most important need contributing to (first-contact) providers perceiving mHealth to be useful was their self-reported lack of capacity to provide adequate care [36, 38–40, 47, 52]. Furthermore, (first-contact) providers reported that they needed support in order to follow guidelines [50, 54, 55] and that mHealth could contribute to overcoming barriers to accessing supervision [37, 40–42, 47, 51]. Also for (specialized) providers, several need factors contributed to the perceived usefulness of the intervention, including, for example, an over-burdening workload [20, 36, 38–41, 43, 45, 47, 50] and a lack of adequate referral and transport systems [38, 46, 47].

Studies did not report that the needs of patients and (first-contact) providers influenced their perceived ease of use. However, specialized providers found mHealth easier to use in the context of certain disease conditions, such as acne, herpes simplex, Kaposi’s sarcoma, and flame burns in dermatology, than in others (scald burns, thickness wounds, and atopic dermatitis) [36, 43] and easier for certain diagnostic and treatment tasks (sharing feedback with patients, continuous clinical follow-ups) than for others (conducting physical examinations, special tests, and probing for additional information) [43, 47, 49, 51].

#### Enabling resources

For patients, unsurprisingly, the two most important enabling resources necessary for a mHealth intervention to be (perceived to be) useful were access to mobile phones [37, 45, 46, 50, 53–55] (also possible through borrowing [46, 50]) and the availability of a functioning stable telecommunications network [20, 36, 39, 42, 45, 47, 48, 52, 55]. Other enabling resources were assurance of privacy [43, 48], support from partners/relatives [51, 55], reduced costs of travel, and reduced time away from home or work [24, 50–53].

Enabling resources for first-contact providers were access to basic infrastructure, such as electric power and functioning medical technologies [49, 52], ambulance services and good roads [46, 47], as well as the affordability of telecommunication services and other operating costs [50, 54, 55] (see Table 2). For (specialized) healthcare providers, the most important enabling resources were a tolerable additional workload [40, 46, 47], the use of financial incentives [47, 55], and the availability of policy guidelines regarding data protection, phone usage, etc. [40, 43, 45, 52].

Enabling resources influencing patients’ perceived ease of use of mHealth interventions included the durability and portability of mobile phones [37, 55] and the low complexity of the technology, for example, short message service (SMS) and icons [53, 55]. The same enabling resources — easy portability and operability [36, 39], using technologies from basic SMS to smartphones, built-in camera, and battery-saving apps [41, 43, 45, 52] — were also found to be associated with the perception among healthcare providers that mHealth was easy to use.

## Discussion

### Summary of main findings

This is the first realist review of mHealth interventions for patients with NCDs in SSA countries. It shows on the basis of a wide range of included studies how, for whom, and in what circumstances mHealth interventions contribute to improved access to (specialized) care for patients with NCDs in SSA. The review did not focus on specific interventions, specific diseases, or specific providers. Instead, it adopted a middle-range perspective to identify how contextual factors influence the outcome of mHealth interventions in terms of improved access to care; in other words, how to identify C-M-O relationships.

Our framework for understanding mHealth interventions illustrates the causal mechanisms that explain how, for whom, and in what circumstances mHealth interventions facilitate access to care (see Fig. 2). As to how mHealth interventions facilitate access to care, a mHealth intervention will ultimately contribute to improved access to care only if it is perceived to be useful and easy to use. The framework therefore shows that predisposing characteristics and needs of patients and healthcare providers as well as enabling resources influence the perceptions of patients and providers that mHealth interventions are useful and easy to use.

Considering for whom or how patients and provider characteristics influence mHealth interventions, the reviewed studies revealed that a positive attitude toward the mobile technology and the ability to communicate in a common language were the most important predisposing characteristics of patients and providers contributing to the perception that mHealth was useful and easy to use. In addition, needs of patients and providers, such as a high perceived burden of disease (e.g., in cases of reduced mobility) and the perceived lack of capacity of first-contact providers to provide adequate care, influenced the perceived usefulness and ease of use.

Furthermore, studies reported that certain circumstances of enabling resources, such as the availability of a stable communications network, accessible maintenance services, and regulatory policies (e.g., on data protection), contribute to the perception of patients and providers that mHealth interventions are useful and easy to use.

### Strengths and implications for policy makers and program managers

This review has several strengths. Following a realist methodology, it has included a wider scope of evidence than previous reviews [6, 10], and it has focused on the policy-relevant questions of how, for whom, and in what circumstances mHealth interventions facilitate access to care. The framework presented in Fig. 2 and the more specific context factors summarized in Table 2 have major implications for policy makers and program managers.

Firstly, given that predisposing characteristics of patients and providers influence the success of mHealth interventions, it is important that these factors are taken into account during the planning stages prior to the introduction of a new mHealth intervention. For example, program managers should consider evaluating the cultural and social acceptance among patients and providers to use the mobile technology when selecting a particular setting for the intervention. In particular, healthcare providers should be recruited who are enthusiastic and motivated to use mHealth as part of their job. Furthermore, interventions should be designed in such a way that patients, providers, and technical support will be able to communicate in a common language; otherwise, interventions are unlikely to be perceived to be useful and easy to use.

Secondly, and similar to the first point, it is important for policy makers and program managers to consider the specific needs of patients and (first-contact) providers to access (specialized) healthcare providers when preparing for the introduction of an mHealth intervention. For example, mHeath interventions will be particularly useful for severely ill patients or patients who face barriers to access (specialized) care, e.g., because they have difficulties in walking. Similarly, those (first-contact) providers who have a particular need for advice and supervision for treating certain groups of patients will perceive mHealth to be particularly useful. In addition, the influence of need factors on the perceived ease of use of (specialized) providers should be considered when preparing the introduction of an mHealth intervention, e.g., that mHealth is better for sharing feedback and continuous follow-up than for special tests and for probing for additional information [43, 47, 49].

Thirdly, policy makers and program managers have to be aware that the availability of enabling resources is essential for the successful implementation of an mHealth intervention. Enabling resources include, for example, easy access to mobile phones/devices, a stable and accessible communications network, and access to basic infrastructural resources, such as roads and ambulance services, which are necessary for mHealth supported referral systems [20, 36, 37, 39, 41, 42, 45–48, 50, 52–55]. Furthermore, policies on data protection and policies limiting the extra workload of mHealth interventions for professionals, possibly providing additional financial incentives, can support the sustained use of mHealth. See the checklist for policy guidance in Table 3.

**Table 3:** A checklist for guiding the selection, development, implementation, evaluation, and policies regarding mHealth for treatment and care of non-communicable diseases in sub-Saharan Africa

Patient context factors
• The personal characteristics of patients, which predispose them to utilize the services provided by the intervention. For example: a. Enthusiasm to use mobile phone/device b. Educational/literacy level c. Age (may be more sustainable among middle/older age groups) d. Local content/language of locality e. Cultural and social acceptance • The needs of patients to access the required healthcare services. For example: a. Disease severity and comorbidities b. Barriers to accessing care/information • The necessary enabling (personal and community) resources to facilitate the implementation of the intervention. This includes: a. Access to mobile phone/device *(essential)* b. Stable and accessible communication networks and technology infrastructure *(essential)* c. Convenience and privacy *(essential)* d. Socio-technical support *(essential)* e. Affordable services *(critical)* f. Awareness raising *(for increased participation)*
Provider context factors
• The personal characteristics of healthcare providers, which predispose them to deliver health services through a mHealth intervention. For example: a. Experience and competence b. Positive attitude toward technology c. Basic knowledge of the technology involved d. Fluency in language of locality e. Understandable language of communication among users and technical support team *(software developers)* • The needs of healthcare providers to deliver the required healthcare services. For example: a. Characteristics of disease conditions *(extent, severity)* b. Characteristics of diagnostic and treatment tasks c. Burden of workload d. Adequacy of referral and transport systems • The necessary enabling (personal and community) resources to facilitate the utilization of the intervention. This includes: a. Access to mobile phone/device and stable networks *(in underserved communities)* b. Easy portability and operability *(features, apps, functionalities, etc.)* c. Available basic infrastructural resources *(good roads, ambulance services)* d. Suitability and equivalence to existing/alternative care processes *(attractive)* e. Tolerable burden of workload and incentives *(essential)* f. Maintenance-technical support *(essential)* g. Continuous training and sensitization h. Low operating costs and available funds/logistics i. Policy and regulation *(network/data protection, staff job descriptions, and contracts, etc.)*

### Limitations

This review has a number of limitations. First, it does not answer the question of whether mHealth interventions facilitate improved access to care for patients with NCDs. It therefore does not contribute to the debate of whether mHealth interventions should be scaled up. Second, given that this review included a broad range of studies with various study designs, the inclusion of a specific study’s finding into the review depended on rather subjective judgments. Following guidelines for realist reviews [11, 12, 26, 27], it was necessary to make decisions about whether a study’s findings were relevant for the development of the framework and whether inferences drawn by an original study were sufficiently supported by evidence. Third, despite an extensive literature search and the inclusion of a wide range of studies, the available evidence on mHealth interventions in SSA remains rather limited. Therefore, the contextual factors summarized in Table 2 are rather indicative. It is very likely that there are further predisposing characteristics, enabling resources, and needs that are relevant for explaining how, for whom, and in what circumstances mHealth interventions work beyond those identified in our review. Future research is needed to confirm the theoretical framework developed in this paper and to operationalize some of its categories. For example, concerning the interplay of predisposing characteristics and perceived usefulness (see Table 2), research is needed to confirm that cultural and social acceptance is a predictor of perceived usefulness. This requires an operationalization for measuring cultural and social acceptance and for quantifying its impact on the sustained use of mHealth. Similarly, more research is necessary to better understand the interplay between need and specialized providers’ ease of use. For example, researchers should explore the suitability of mHealth applications for different diseases and concerning different diagnostic and treatment tasks. This could include an assessment of the ease of use of mHealth for sharing feedback with patients with different diseases or different levels of severity, e.g., diabetes versus hypertension or diabetes with and without complications, and the differential effects on health outcomes.

## Conclusions

The implementation of mHealth interventions in SSA has great potential to improve treatment and care for patients with NCDs, but the causal mechanisms explaining why, how, for whom, and in what circumstances these interventions work used to be unexplored. Our realist review shows that the contribution of mHealth interventions to improved treatment and care for patients with NCDs is that they facilitate (remote) access to previously unavailable — and often specialized — services. In addition, our framework for understanding mHealth interventions illustrates that predisposing characteristics and needs of patients and healthcare providers as well as the availability of enabling resources in the community influence the perceptions of patients and providers that mHealth interventions are useful and easy to use — and these perceptions are essential for the successful implementation of an mHealth intervention.

The implication of these findings for policy makers and program managers is that predisposing characteristics and needs of patients and providers as well as the necessary enabling resources should be considered during the planning stages prior to the introduction of an mHealth intervention. In addition, researchers would benefit from placing greater attention on the context in which mHealth interventions are being implemented — as the context largely determines the predisposing characteristics and needs of patients and providers as well as the enabling resources — instead of focusing (too strongly) on the technical aspects of these interventions.

## Additional files

Additional file 1: List of databases accessed and the search strategies used. (DOCX 20 kb)
Additional file 2: Theories included or excluded in review. (ZIP 28 kb)
Additional file 3: Description of the included articles providing the information for the synthesis and conclusions in this review. (DOCX 24 kb)

## Abbreviations

C-M-O: Context-Mechanism-Outcome
mHealth: mobile phone-based health
NCD: non-communicable disease
SSA: sub-Saharan Africa
WHO: World Health Organization
