# Supplementary material for: A realist review of mobile phone-based health interventions for non-communicable disease management in sub-Saharan Africa
Source: BMC Med. 2017 Feb 6;15:24. doi: 10.1186/s12916-017-0782-z (PMC5292812; doi:10.1186/s12916-017-0782-z)
Supplement: Additional file 1: — List of databases accessed and the search strategies used. (DOCX 20 kb) [file 12916_2017_782_MOESM1_ESM.docx]

*Additional file 1*: **Search strategy for four databases as at May 31, 2015**

| **Search** | **PubMed QUERY** | **Items found** |
| --- | --- | --- |
| #1 | mHealth[MeSH Terms] | [16186](http://www.ncbi.nlm.nih.gov/pubmed/?cmd=HistorySearch&querykey=4) |
| #2 | Sub-Saharan Africa [MeSH Terms] | [144985](http://www.ncbi.nlm.nih.gov/pubmed/?cmd=HistorySearch&querykey=6) |
| #3 | #1 AND #2 | [150](http://www.ncbi.nlm.nih.gov/pubmed/?cmd=HistorySearch&querykey=7) |
| #4 | (((("mHealth") OR ("m-health") OR ("mobile health") OR ("telemedicine") OR (“mobile device”) OR (“mobile phone”) OR (“cellular phone”) OR ("phone") OR ("text messaging") OR ("SMS") OR (“messaging”) OR (“call”) OR ("App") OR ("Handheld Computers") OR (tele*)))) | [110330](http://www.ncbi.nlm.nih.gov/pubmed/?cmd=HistorySearch&querykey=8) |
| #5 | ((((“sub-Saharan Africa”) OR (“Africa”) OR (“developing world”) OR (“developing count*”) OR (“low income”) OR (“middle income”) OR (“low and middle income”) OR (“LMICs”) OR (“LAMICs”) OR (“low resource”) OR (“resource poor”) OR (“limited resource”) OR (“tropical”)))) | [256139](http://www.ncbi.nlm.nih.gov/pubmed/?cmd=HistorySearch&querykey=9) |
| #6 | #4 AND #5 | [2427](http://www.ncbi.nlm.nih.gov/pubmed/?cmd=HistorySearch&querykey=10) |
| #7 | #3 OR #6 | [2491](http://www.ncbi.nlm.nih.gov/pubmed/?cmd=HistorySearch&querykey=11) |

|  |  |  |
| --- | --- | --- |
|  | **WEB OF SCIENCE QUERY** |  |
| #1 | TS=(mHealth OR m-health OR mobile health OR telemedicine OR mobile device OR mobile phone OR cellular phone OR phone OR text messaging OR SMS OR messaging OR call OR App OR Handheld Computers OR tele*)  Indexes=SCI-EXPANDED, SSCI, A&HCI Timespan=All years | [903,138](http://apps.webofknowledge.com/summary.do?product=WOS&doc=1&qid=5&SID=N1hlo7dZdBohl6gxZF1&search_mode=AdvancedSearch&update_back2search_link_param=yes) |
| #2 | \|  \| TS=((“sub-Saharan Africa”) OR (“Africa”) OR (“developing world”) OR (“developing count*”) OR (“low income”) OR (“middle income”) OR (“low and middle income”) OR (“LMICs”) OR (“LAMICs”) OR (“low resource”) OR (“resource poor”) OR (“limited resource”) OR (“tropical”))  Indexes=SCI-EXPANDED, SSCI, A&HCI Timespan=All years \| \| --- \| --- \| | [435,172](http://apps.webofknowledge.com/summary.do?product=WOS&doc=1&qid=6&SID=N1hlo7dZdBohl6gxZF1&search_mode=AdvancedSearch&update_back2search_link_param=yes) |
| #3 | TS=(("non-communicable disease") OR ("noncommunicable disease") OR ("chronic disease") OR (“NCDs”)OR ("diabetes") OR ("cardiovascular disease") OR ("respiratory tract") OR ("neoplasms") OR ("cancer") OR (“congenital”) OR (“digestive”) OR (“respiratory”) OR (“hypertension”) OR (“sickle cell”) OR (“oral”) OR (“ophthalmology”) OR (“eye”) OR (“ear”) OR (“mental”) OR (“psychiatry”) OR (“depression”))  Indexes=SCI-EXPANDED, SSCI, A&HCI Timespan=All years | [3,809,025](http://apps.webofknowledge.com/summary.do?product=WOS&doc=1&qid=8&SID=N1hlo7dZdBohl6gxZF1&search_mode=AdvancedSearch&update_back2search_link_param=yes) |
| #4 | #3 AND #2 AND #1  Indexes=SCI-EXPANDED, SSCI, A&HCI Timespan=All years | [1,836](http://apps.webofknowledge.com/summary.do?product=WOS&doc=1&qid=9&SID=N1hlo7dZdBohl6gxZF1&search_mode=CombineSearches&update_back2search_link_param=yes) |
|  |  |  |
|  | **COCHRANE QUERY** |  |
| #1 | MeSH descriptor: [Telemedicine] explode all trees | 1302 |
| #2 | MeSH descriptor: [Africa South of the Sahara] explode all trees | 4032 |
| #3 | #1 and #2 | 3 |
| #4 | (("mHealth") or ("m-health") or ("mobile health") or ("telemedicine") or ("mobile device") or ("mobile phone") or ("cellular phone") or ("phone") or ("text messaging") or ("SMS") or ("messaging") or ("call") or ("App") or ("Handheld Computers") or (tele*)) | 18107 |
| #5 | (("sub-Saharan Africa") or ("Africa") or ("developing world") or ("developing count*") or ("low income") or ("middle income") or ("low and middle income") or ("LMICs") or ("LAMICs") or ("low resource") or ("resource poor") or ("limited resource") or ("tropical")) | 14841 |
| #6 | #4 and #5 | 1504 |
| #7 | (("non-communicable disease") or ("noncommunicable disease") or ("chronic disease") or ("NCDs") or ("diabetes") or ("cardiovascular disease") or ("respiratory tract") or ("neoplasms") or ("cancer") or ("congenital") or ("digestive") or ("respiratory") or ("hypertension") or ("sickle cell") or ("oral") or ("ophthalmology") or ("eye") or ("ear") or ("mental") or ("psychiatry") or ("depression")) | 378526 |
| #9 | #7 and #6 | 1186 |
| #10 | #9 or #3 | 1188 |
|  |  |  |
|  | **GOOGLE SCHOLARS QUERY** |  |
|  | (("mHealth") OR ("mhealth") OR ("mobile health") OR ("telemedicine") OR (“mobile device”) OR (“mobile phone”) OR (“cellular phone”) OR ("phone") OR ("text messaging") OR ("Handheld Computers")) AND ((“sub-Saharan”) OR (“Africa") OR ("LMIC)) | 684 |
