# Supplementary material for: A realist review of mobile phone-based health interventions for non-communicable disease management in sub-Saharan Africa
Source: BMC Med. 2017 Feb 6;15:24. doi: 10.1186/s12916-017-0782-z (PMC5292812; doi:10.1186/s12916-017-0782-z)
Supplement: Additional file 2: — Theories included or excluded in review. (ZIP 28 kb) [file 12916_2017_782_MOESM2_ESM.zip › Additional file 4_Theories include or excluded in reviewR1.docx]

**Table S3**

| **Theories considered for this review and grounds of inclusion/exclusion** | |
| --- | --- |
| **Theory/Model** [ref. in article] | **Brief description [Inclusion/Exclusion]** |
| 1. Middle-Range Theory of Self-Care of Chronic Illness [28] | Describes self-care as the process of maintaining health with health promoting practices within the context of the management required of a chronic illness.  [***Excluded*:** this theory treats access to care not necessarily as an outcome/output measure but rather a factor that can impact on self-care] |
| 1. Theory of Reasoned Action/Theory of Planned Behaviour [29] | Predicting and understanding of individual behaviour as a function of a formed intention based on beliefs, norms, attitudes and self-efficacy.  [***Exclusion:*** Technology acceptance model was preferred and deemed fit because it has been based on reasoned action approach and directly relate to the assessment of technology-based interventions] |
| 1. Rosenstock’s Health Belief Model [30] | Considers a person’s actions to treat and prevent disease because of the individual’s perceived susceptibility to disease, threat of disease, costs-benefits and cues to action.  [***Excluded:*** the model here concentrates partly on disease prevention which falls outside the scope of this review] |
| 1. Andersen’s Behavioural Model of Health Services Utilization [31, 32] | According to Andersen’s original model, peoples’ decisions to use (or access) healthcare services are determined by three main factors: (1) predisposing characteristics (e.g. age, health beliefs), (2) enabling resources (e.g. availability of providers) and (3) need (e.g. burden of disease).  [***Included:*** it could potentially provide insights into the mechanisms linking contextual and individual level factors with improved access to care] |
| 1. Young’s Choice-Making Model [33] | The model considers that individual’s health service choice is based on perceived gravity of illness, the knowledge of a home treatment, faith in treatment and accessibility of treatment.  [***Excluded:*** access to treatment (care) is considered an important influential factor on health care utilization and not necessarily an outcome/output] |
| 1. Davis’s Technology Acceptance Model [34, 35] | Posits that the use and acceptance of technology is determined by two factors: *perceived usefulness* and *perceived ease of use.* Thus, health professionals will perceive a technology to be useful if they belief that it will help them to do a better job; and they will perceive a technology to be easy to use if they believe that it can be used without effort.  [***Included:*** provides additional insights into mechanisms that are important for explaining individual’s behaviour on information technology and information systems] |
