# Supplementary material for: A realist review of mobile phone-based health interventions for non-communicable disease management in sub-Saharan Africa
Source: BMC Med. 2017 Feb 6;15:24. doi: 10.1186/s12916-017-0782-z (PMC5292812; doi:10.1186/s12916-017-0782-z)
Supplement: Additional file 3: — Description of the included articles providing the information for the synthesis and conclusions in this review. (DOCX 24 kb) [file 12916_2017_782_MOESM3_ESM.docx]

**Additional file 3**

**Table S1: Key characteristics of included studies for synthesis**

| Ref. No. | **Author (year)** | **Title** | **Country** | **Intervention** | **Healthcare setting** | **Study design** | **Disease indication** | **Modality of interaction** | **User -participants** |
| --- | --- | --- | --- | --- | --- | --- | --- | --- | --- |
| 36 | Azfar, R. S., et al. (2014) | Reliability and Validity of Mobile Teledermatology in Human Immunodeficiency Virus-Positive Patients in Botswana A Pilot Study. | Botswana | (inter-national) mobile phone teledermatology diagnosis and management evaluation | Hospital Clinics Centers | Cross-sectional pilot cohort | dermatology | provider-to-provider | 4 providers  76 patients cases |
| 20 | Azfar, R. S., et al. (2011) | HIV-positive patients in Botswana state that mobile teledermatology is an acceptable method for receiving dermatology care. | Botswana | (inter-national) mobile teledermatology evaluation of patients with skin or mucosal complaints | Hospital Clinics Centers | Cross-sectional survey | dermatology | patient-to-provider | 75 patients |
| 37 | Chindo, L. (2013) | Mobile phone use in Cameroon: an increasingly useful tool for the follow-up of children with Burkitt lymphoma. | Cameroon | (in-country) use of mobile phone to contact patients’ parents | Hospital | Record review (letter) | Burkitt lymphoma (BL) | patient (parent proxy)-to-provider | 285 patient cases |
| 38 | Fiander, A., et al. (2013) | Results from 2011 for the transportMYpatient program for overcoming transport costs among women seeking treatment for obstetric fistula in Tanzania. | Tanzania | (in-country) mobile phone technology to transfer funds to cover patients’ transport costs | Hospital | Record review | obstetric fistula | provider-to-provider | 286 patients 253 health workers |
| 39 | Fruhauf, J., et al. (2013) | Mobile teledermatology in sub-Saharan Africa: a useful tool in supporting health workers in low-resource centres. | Uganda | (inter-national) smartphone-based remote expert diagnosis of dermatology cases | Health Center | Mixed methods | skin diseases: inflammatory, infectious, neoplastic, autoimmune and others | provider-to-provider | 72 patients 4 health workers |
| 40 | Greisman, L., et al. (2014) | Feasibility and cost of a medical student proxy-based mobile teledermatology consult service with Kisoro, Uganda, and Lake Atitlan, Guatemala. | Uganda | (inter-national) smartphone-based dermatology consult service | Hospital | Mixed methods | skin diseases: Biopsy | provider-to-provider | 57 patients 1 student proxy |
| 41 | Holeman, I., et al. (2014) | Mobile health for cancer in low to middle income countries: priorities for research and development. | Malawi | (in-country) SMS exchange between CHWs and a nurse at a district hospital | Hospital | Commentary | cancers, HIV or Tuberculosis. | provider-to-provider | 75 health workers |
| 42 | Kingue, S., et al. (2013) | Efficiency of an intervention package for arterial hypertension comprising telemanagement in a Cameroonian rural setting: The TELEMED-CAM study. | Cameroon | (in-country) GSM mobile telephone communication between staff at health centers and the telecare center | Health Center | Prospective interventional study | hypertension | provider-to-provider | 5 cardiologists  268 patients |
| 43 | Kiser, M., et al. (2013). | Photographic assessment of burn wounds: a simple strategy in a resource-poor setting. | Malawi | (in-country) cell phone-based cameras used for photographing wounds for experienced clinician's TBSA estimations and examination. | Hospital | Cohort study | burn injury | provider-to-provider | 2 burn physicians/ clinicians  39 patients |
| 53 | Kivuti-Bitok, L. W., et al. (2012) | Self-reported use of internet by cervical cancer clients in two National Referral Hospitals in Kenya. | Kenya | (in-country) use of eHealth tools in cervical cancer management | Hospital | Cross sectional descriptive survey | cervical cancer | patient-to-(system) provider | 199 patients |
| 52 | Littman-Quinn, R., et al. (2013) | Implementation of m-health applications in Botswana: telemedicine and education on mobile devices in a low resource setting. | Botswana | (inter-national/in-country) smartphone-based management of complex patient cases by collecting pertinent clinical history and associated images for specialist consultation | public private academic non-profit institutions | - | women's health (cervical cancer) radiology oral medicine dermatology | provider-to-provider | 24 clinicians  643 cases |
| 45 | Ndlovu, K., et al. (2014) | Scaling up a Mobile Telemedicine Solution in Botswana: Keys to Sustainability. | Botswana | (inter-national/in-country) smartphone-based management of complex patient cases by collecting pertinent clinical history and associated images for specialist consultation | public private academic non-profit institutions | Pilot study | women's health (cervical cancer) radiology oral medicine dermatology | provider-to-provider | 27 clinicians  696 cases |
| 46 | Odigie, V. I., et al. (2012) | The mobile phone as a tool in improving cancer care in Nigeria. | Nigeria | (in-country) phone-based consultation about medical care | Hospital | Structured interview | cancer | patient-to-provider | 1176 patients  1 oncologist |
| 47 | Opoku, D., et al. (2015) | Healthcare Professionals' Perceptions of the Benefits and Challenges of a Teleconsultation Service in the Amansie-West District of Ghana. | Ghana | (in-country) teleconsultation for treating patients in times of difficulties | Hospital | Qualitative study | health-related outcomes | provider-to-provider | 8 health workers  3 healthcare professionals |
| 48 | Osei-tutu, A., et al. (2013) | Mobile teledermatology in Ghana: sending and answering consults via mobile platform. | Ghana | (in-country and inter-national) mobile telephone-based images and data collected for teledermatological diagnosis | Clinic | Pilot study | dermatology | provider-to-provider | 5 dermatologists  34 patients |
| 54 | Pastakia, S. D., et al. (2011) | The evolution of diabetes care in the rural, resource-constrained setting of western Kenya. | Kenya | (in-country) cell phone-based support to patients on nonclinical days | Health Center | - | diabetes | patient-to-provider | 2100 patients  >2 healthcare professionals |
| 49 | Qin, R., et al. (2013) | Reliability of a telemedicine system designed for rural Kenya. | Kenya | (in-country) store-and-forward mobile telemedicine system to collect and send vital statistics | Health Center | Modified intraobserver concordance study | NCDs: diabetes, hypertension | provider-to-provider | 102 patients  1 nurse  1 CHW |
| 55 | Rotheram-Borus, M. J., et al. (2012) | Diabetes buddies: peer support through a mobile phone buddy system. | South Africa | (in-country) mobile phone-based peer support intervention among women in resource-poor settings | Clinic | Pilot study | diabetes | patients-to-provider | 22 patients  3 nursing sisters |
| 50 | Stewart, A. N., T.; Eales, C.; Shepard, K.; Becker, P.; Veriawa, Y. (2005) | Adherence to cardiovascular risk factor modification in patients with hypertension. | South Africa | (in-country) a once-a-month telephone call by healthcare practitioners to both the patients and a member of their famlies. | Hospital | Randomized controlled trial | hypertension | patient-to-provider | 83 patients  1 physiotherapist |
| 51 | Temmingh, H., et al. (2013) | The evaluation of a telephonic wellness coaching intervention for weight reduction and wellness improvement in a community-based cohort of persons with serious mental illness. | South Africa | (in-country) telephonically delivered life style and wellness program by lifestyle coaches | Hospital | Cohort study | mental health | patient-to-provider | Lifestyle coaches  761 participants |
